# Quantification of verbascoside in medicinal species of *Phlomis* and their genetic relationships

**DOI:** 10.1186/2008-2231-22-32

**Published:** 2014-03-20

**Authors:** Parisa Sarkhail, Marjan Nikan, Pantea Sarkheil, Ahmad R Gohari, Yousef Ajani, Rohollah Hosseini, Abbass Hadjiakhoondi, Soodabeh Saeidnia

**Affiliations:** 1 Pharmaceutical Sciences Research Center, Tehran University of Medical Sciences, Tehran, Iran; 2 Medicinal Plants Research Center, Faculty of Pharmacy, Tehran University of Medical Sciences, Tehran, Iran; 3 Division of Pharmacy, College of Pharmacy and Nutrition, University of Saskatchewan, Saskatoon, Canada; 4 Institute of Medicinal Plants (IMP), Iranian Academic Centre for Education, Culture and Research (ACECR), Karaj, Iran; 5 Department of Toxicology and Pharmacology, Faculty of Pharmacy, Tehran University of Medical Sciences, Tehran 1417614411, Iran; 6 Department of Pharmacognosy, Faculty of Pharmacy, Tehran University of Medical Sciences, Tehran 1417614411, Iran

**Keywords:** *Phlomis*, RAPD, Verbascoside, Quantification, TLC scanner

## Abstract

**Background:**

The genus *Phlomis* (Lamiaceae) is introduced by its valuable medicinal species, of which 17 species are growing wildly and ten of them are exclusively endemic of Iran. The main phytochemical characteristic of this genus is presence of iridoid glycosides including ipolamide, auroside, lamiide and also phenylethanoids such as verbascoside (acetoside) found in Lamiales order.

Due to the broad range of biological and pharmacological activities of verbascoside and lack of any report on quantification of this compound within Iranian species of *Phlomis*, we conducted a research to achieve two main goals, finding a genetic biodiversity by RAPD (Randomly Amplified Polymorphic DNA), as well as detecting and quantifying verbascoside in nine species of *Phlomis* growing wildly in Iran.

**Results:**

The results showed that various samples of *P.olivieri* possess different genetic distances from each other. Also, various species of *P.olivieri* display close relationships to *P.anisodonta* and *P. persica*. Phytoanalysis of *Phlomis* species by means of TLC scanner using verbascoside as a phytochemical marker showed that the highest concentration of verbascoside was found in *P. anisodonta*, however, *P. bruguieri* and *P. olivieri* (from Mazandaran) were in the second and third places. Interestingly, the lowest concentration of verbascoside was detected in *P. olivieri* (from Azerbayjan), exhibiting the effect of various growing areas and conditions on the measured levels of this compound.

**Conclusions:**

verbascoside can be found in various species of Iranian *Phlomis*, of which *P. anisodonta*, *P. bruguieri* and *P. olivieri* might be the best choices. In addition, although the concentration of verbascoside in these plants may be affected by the growing areas and conditions, there are a good agreement between genetic relations and verbascoside levels.

## Background

The genus *Phlomis* (Lamiaceae) is introduced by its valuable medicinal properties. Seventeen species of *Phlomis* are growing wildly and ten of them are exclusively endemic of Iran [1,2]. The medicinal importance of *Phlomis* species (called: Gush-e Barreh in Persian language) had described by Dioscorides (first century B.C.) in “*De MateriaMedica*”, and these plants have been employed in herbal medicine for respiratory tract disorders or wound healing till date [3]. However, a number of *Phlomis* species have been consumed in folk medicine as antitussive remedies, and also for gastrointestinal complains, tonic, sedative, carminative and astringent agents a well [4].

A survey on phytochemical characteristics of this genus revealed that *Phlomis* species are rich in iridoids, flavonoids, terpenoids, phenolic compounds and their glycosides, which are attributed to various biological and pharmacological effects of them including antinociceptive, antioxidant, antimicrobial and anti-diabetic effects [3-6]. The main phytochemical characteristics of this genus are presence of the iridoid glycosides including ipolamide, auroside, lamiide and also phenylethanoids such as verbascoside (acetoside), which is a caffeic acid sugar ester and can be found in plant species of Lamiales order [7-9].

Regarding to remarkable role of verbascoside in generation of a broad range of biological and pharmacological activities [10-12] and lack of any report on quantification of this compound within Iranian species of *Phlomis*, we tried to conduct a research to achieve two main goals, detection and quantification of verbascoside in nine species of *Phlomis* growing wildly in Iran, as well as finding a genetic biodiversity among them by RAPD analysis (Randomly Amplified Polymorphic DNA) appraising to the presence of a chemotaxonomic marker, verbascoside, whether or not it will be in agreement with phylogenetic cladogram.

## Methods

### Sample collection

Locations, altitudes and collection periods of the plant materials used in this study are given in Table 1. The plants were identified by Mr. Yousef Ajani from Institute of Medicinal Plants, Karaj, Iran. The voucher specimens have been deposited at two Herbariums located at Faculty of Pharmacy, Tehran University of Medical Sciences, and Institute of Medicinal Plants (IMP), Iranian Academic Centre for Education, Culture and Research (ACECR) in Iran. Plant materials were dried in shadow and the leaves of the plants were separated from the stem, and were ground to powder in porcelain mortars with liquid nitrogen. Then, the powdered plant material was used for DNA extraction.

**Table 1:** Locations, altitudes and collection periods of the plant materials used in RAPD analysis

**Number**	**Herbarium No.**	**Species**	**Location**	**Altitude (m)**	**Date**
**1**	1612	*P. olivieri*	Marivan to paveh, 51 km a paveh 35°14’27.7”N, 46°11’45.6”E	2448	07, 2011
**2**	1611	*P. persica*	Sanandaj to marivan, 108 km a marivan 35°24’53.9”N, 46°53’12.5”E	1766	07, 2011
**3**	1557	*P .rigida*	Sanandaj to marivan,between sheikh attar and baghan 35°30’54.0”N, 46°27’32.5”E	1598	07, 2011
**4**	1582	*P.kurdica*	Sanandaj to marivan, 41 km a sanandaj 35°22’51.7”N, 46°42’46.7”E	1595	07, 2011
**5**	1610	*P.persica*	Olalan region, 5 km a kargabad to salavatbad 35°17’13.6”N, 47°09’42.3”E	2218	07, 2011
**6**	1581	*P. bruguieri*	Sanandaj to marivan,in the beginning of the road 35°22’35.4”N, 47°00’11.5”E	1519	07, 2011
**7**	1580	*P. anisodonta*	Marivan to paveh between dezli and hanigarmohalleh 35°16’39.4”N, 46°11’11.2”E	2246	07, 2011
**8**	1648	*P. caucasica*	Ahar; Khoy to qotur, 45 km a qotur, 35° 22’51.7”N, 46°42’ 46.7”E	-	07, 2011
**9**		*P. olivieri*	Shabestar (details are not available)	-	07, 2010
**10**	1631	*P. olivieri*	Azerbayjan; Khoy to qotur, 45 km a qotur, 35°22’51.7”N, 46°42’46.7”E	1297	06, 2011
**11**	_	*P. olivieri*	Tabriz (details are not available)	-	07, 2010
**12**	1634	*P. anisodonta*	Mazandaran, 5 km after pol-e zanguleh toward yush, 36°12’0.3.3”N, 51°20’50.7”E	2558	06, 2011
**13**	6532	*P. persica*	North of Iran (details are not available)	-	07, 2002
**14**	6534	*P. olivieri*	Mazandaran (details are not available)	-	07, 2001
**15**	6531	*P. anisodonta*	North of Iran, pol-e- zangule	-	07, 2002

### DNA extraction

Genomic DNA(s) were extracted from the plant materials using a modified method, which was already described [13]. Approximately 150 mg of each plant sample was frozen in liquid nitrogen (in 2-ml Eppendorf tubes). 500 ml of DNA extraction buffer (contains, 2% CTAB {cetyl trimethylammonium bromide}, 100 mM TrisHCl (pH = 8), 20 mM EDTA {ethylene diamine tetra acetic acid}, 1.4 M NaCl, 0.2% 2-mercaptoethanol and 4% PVP {polyvinylpyrrolidone}) was added to each Eppendorf tube and mixed well. The mixture was incubated at 65˚C in a water bath for 60 min with intermittent shaking at 5 to 10 min intervals. The mixture was mixed with equal volume of phenol: cloroform: isoamylalchol (25:24:1), and centrifuged at 13000 × g for 10 min at 24°C. The supernatant was transferred into a new 1.5-ml tube and 800 μl cold isopropanol (from freezer) was added and inverting until thoroughly mixed and placed in the freezer (-20°C) for 20 min. The mixture centrifuged at 13000 × g for 5 min at 4°C. The supernatant was removed and the precipitate was kept at room temperature for 15 min and then, mixed gently with 300 μl ammonium acetate (7.5 M) for 20 min at room temperature. After centrifugation at 13000 × g for 10 min at 4°C, the supernatant was removed and 600 μl of ethanol (70%) was added then centrifuged at 6000 × g for 10 min at 4°C. The DNA was pelleted by centrifugation and the ethanol was poured off, the DNA was allowed to air-dry before being dissolved in 200 μlof TE buffer.

### Primers

Thirty RAPD primers (ten-mers) were purchased from two companies: Operon Technologies, Alameda, California, USA and Cinnagen, Tehran, Iran. The primers were tested for amplification in a preliminary study, because in RAPD analysis, some primers will work and some may not. In preliminary study, quickly screening of three sets of primers, which previously used successfully for RAPD analysis of other Labiatae species [14,15], was performed using some samples of *Phlomis,* and then those which were giving good profiles for analysis of a large number of samples were selected. Some of the primers were chosen for further analysis (Table 2) based on their ability to produce distinct and polymorphic amplified products within the samples [14]. As a matter of fact, RAPD does not need any particular knowledge of the DNA sequence of the target organism and the amplification is randomly performed. Because the identical 10-mer primers can (or cannot) amplify a segment of DNA, relating to the positions, which are complementary to the primers’ sequence. For instance, when a mutation has occurred in one of the samples (DNAs) just at the site that was already complementary to the primer, a PCR product would not be produced, therefore a different pattern of amplified DNAs might be observed.

**Table 2:** Sequencing primers used for RAPD analysis together with total number of amplified fragments and the polymorphism percentage

**Primer**	**Sequences 5’ to 3’**	**Total bands**	**Polymorphism percentage**
**Zo1**	GGT-CGG-AGA- <A>	11	100
**Zo2**	TCG-GAC-GTG- <A>	8	100
**Zo3**	AGA-CGT-CCA- <C>	7	87
**Zo4**	GGA-AGT-CGC- <C>	13	88
**Zo5**	AGT-CGT-CCC- <C>	10	100
**Zo6**	CTG-CAT-CGT-- <G>	11	100
**Zo7**	GAA-ACA-CCC- <C>	9	90
**Zo8**	TGT-AGC-TGG- <G>	7	98
**Zo9**	ACG-CGC-ATG- <T>	13	78
**Zo10**	GAC-GCC-ACA- <C>	10	96
**Zo11**	ACC-AGG-TTG- <G>	13	100
**Zo12**	AAT-GGC-GCA- <G>	16	100
**Zo13**	CAC-TCT-CCT- <C>	12	100
**Zo14**	GAA-TCG-GCC- <A>	13	89
**Zo15**	CTG-ACC-AGC- <C>	7	99
**Zo16**	GGG-AGA-CTA- <C>	8	98
**Zo17**	ACA-ACG-CGA- <G>	10	89
**Zo18**	CCG-CCT-AGT- <C>	13	100
**Zo19**	GGA-GGA-GAG- <G>	7	100
**Zo20**	TCA-TCC-GAG- <G>	5	100
**Zo21**	CAG-AAG-CCC- <A>	9	90
**Zo22**	AAG-GCG-GCA- <G>	10	96
**Zo23**	CAG-CGA-CAA- <G>	6	100
**Zo24**	TGG-AGA-GCA- <G>	7	100
**Z025**	ACA-TGC-CGT- <G>	4	100
**Zo26**	CTG-GGG-CTG- <A>	10	100
**Zo27**	TGA-CGG-AGG- <T>	9	98
**Zo28**	TCT-CCG-CCC- <T>	9	78
**Zo29**	TGC-CCA-GCC- <T>	13	90
**Zo30**	AAA-GTG-CGG- <G>	7	96

### RAPD assay

Polymerase chain reactions (PCR) with single primer were carried out in a final volume of 20 μl containing 20 ng template DNA, 20 ng of primer (0.5 to 1 μl), 6 μl of RNase-free water and 10 μl of Taq PCR Master Mix kit (includes 1.5 mM MgCl_2_, 125 units of TaqDNA Polymerase, and 200 μM each dNTP), purchased from Qiagen, USA. Amplification was performed in a Primus 25 (Peqlab, Germany) thermal cycler, programmed for a preliminary 3 min denaturation step at 94°C, followed by 44 cycles of denaturation at 94°C for 30 s, annealing at 36 + 4˚C/ 30 s and extension at 72°C for 1 min, finally at 72°C for 2 min for amplification. PCR products (alongside the negative control and GelPilot DNA Mulecular Weight Marker: 100 bp) were separated by 1% (w/v) agarose gel electrophoresis for RAPD respectively. Green viewer (4 μl) was used to visualize under UV light (Benchtop 3 UV™ Transilluminator) and photographs were recorded by a Canon digital camera. Data were summarized based on the presence or absence of unique and shared polymorphic bands from the photographs. Each amplification fragment was detected by approximate size in base pairs. The DNA profiles were scored visually from photographs of the gels. Reproducible bans (observed at least for two times) were considered for analysis. A pair-wise difference matrix between samples was determined for the RAPD data using simple matching coefficient (Ssm) followed by calculation of genetic distances (d) [15]. UPGMA (unweighted pair-group method arithmetic average) was used to construct the dendrogram. UPGMA employed a sequential clustering algorithm, in which genetic distances were used in order to show similarity, and the phylogenetic tree was built in a stepwise manner [16]. Also, the number of unique bands in various samples by each primer is shown in a table as Additional file 1. In this method, the first cluster is built on the pair of plant DNAs with the smallest distance, then following the first cluster is considered as a single composite and the new distance matrix can be calculated as follows:

$$\mathrm{Distance}\left(A,B\right),C=\left(\mathrm{distance}\;\mathrm{AC}+\mathrm{distance}\;\mathrm{BC}\right)$$

Again, a new distance matrix is recalculated using the newly calculated distances and the whole cycle is being repeated. The final step consists of clustering the last Plant DNA sample with the composite of all others.

**Figure 1:**
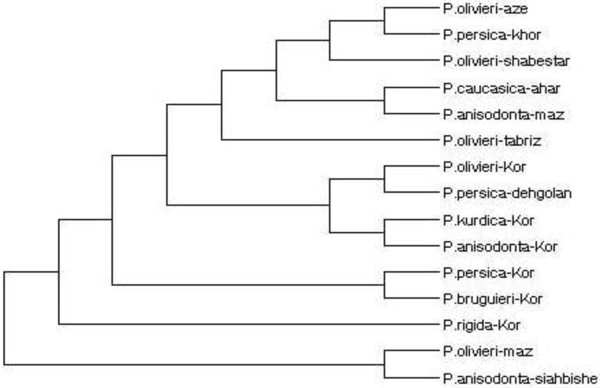
**Cladogram of *Phlomis *samples based on the UPGMA analysis.** (1) *P. olivieri-kor* (Kordestan), (2) *P. persica-kor* (Kordestan), (3) *P. rigida-kor* (kordestan), (4) *P. kurdica-kor* (Kordestan) , (5) *P. persica-dehgolan* (kordestan) (6) *P. bruguieri-kor* (Kordestan), (7) *P. anisodonta-kor* (Kordestan), (8) *P. caucasica* (Ahar), (9) *P. olivieri-shabestar* (Shabestar), (10) *P. olivieri-azer* (Azerbayjan), (11) *P. olivieri-tabriz* (Tabriz), (12) *P. anisodonta-maz* (Mazandaran), (13) *P. persica-khor* (Khorasan), (14) *P. olivieri-maz* (Mazandaran), (15) *P. anisodonta-siahbishe* (Pole zangole).

**Figure 2:**
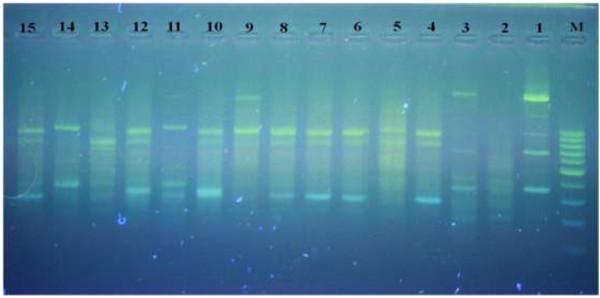
**RAPD profile produced from the primer, Zo 21; M: DNA size marker.** (1) *P. olivieri* (Kordestan), (2) *P. persica* (Kordestan), (3) *P. rigida* (kordestan), (4) *P. kurdica* (Kordestan) , (5) *P. persica* (dehgolan) (6) *P. bruguieri* (Kordestan), (7) *P. anisodonta* (Kordestan), (8) *P. caucasica* (Ahar), (9) *P. olivieri* (Shabestar), (10) *P. olivieri* (Azerbayjan), (11) *P. olivieri* (Tabriz), (12) *P. anisodonta* (Mazandaran), (13) *P. persica* (Khorasan), (14) *P. olivieri* (Mazandaran), (15) *P. anisodonta* (Pole zangole).

The cladogram constructed base on genetic distances, derived from RAPD analysis, shown in Figure 1. The indicated cladogram was designed by the software Dendroscope which is freely available from http://dendroscope.org[17,18]. Also, Figure 2 exhibits a gel electrophoresis of RAPD pattern of the plant DNA samples with primer Zo21.

### HPTLC (High Performance Thin Layer Chromatography) analysis

#### *Reagents and Instruments*

Silica gel 60 F_254_ pre-coated plates (Merck) were used for preliminary TLCs. The spots were detected by spraying anisaldehyde-H_2_SO_4_ reagent followed by heating. 

All the chemicals and reagents used for TLC were purchased from Merck by analytical grades. The instrument for HPTLC was from CAMAG. The TLC scanner was CS-9000, Dual Wavelength, Flying-spot Scanner (Shimadzu).

#### *Sample preparation for TLC scanner*

Dried and powdered leaves of *Phlomis* species (100 g) were extracted twice with MeOH (80%, 1500 ml) in percolator for one week. The combined methanol extracts were evaporated by Rotary Evaporator. Dried crude extract was dissolved in water and the water soluble portion was successively fractionated using dichloromethane, diethyl ether and n-butanol, respectively. The n-butanol layers were combined and concentrated to dryness in vacuo at <45°C. The n-butanol extracts (80 mg/mL) and the standard solutions of verbascoside were prepared in MeOH.

#### *Thin - layer chromatography*

The plates were pre washed with methanol and dried for 24 h at room temperature. Before use they were activated at 120°C for 30 min. The activated plates were manually spotted with 1 μL aliquots of the solutions. The mobile phase (ethylacetate: water: formic acid, 10:3:2) was used per development. Plates were developed to a distance of 15 mm in chromatographic chamber. Then the plates were dried in a current of air by means of an air dryer. Densitometer scanning was then performed at λmax = 234 nm. The radiation source was a deuterium lamp emitting a continuous UV spectrum between 200-370 nm. Each analysis was repeated five times, whilst each track scanned three times, and baseline correction (lowest slope) was used. The start wavelength was 200 nm and the end wavelength was 370 nm. The verbascoside was quantified by densitometric scanning of the developed plate at 270 nm.

### Validation of the method

#### *Linearity of detector response*

The linearity of the TLC method was evaluated by analysis of 4 standard solutions of verbascoside at concentrations 1.2, 0.9, 0.6 and 0.3 mg/mL. The solutions were applied on the same plate. The plate was developed using the above-mentioned mobile phase.

#### *Specificity*

**Figure 3:**
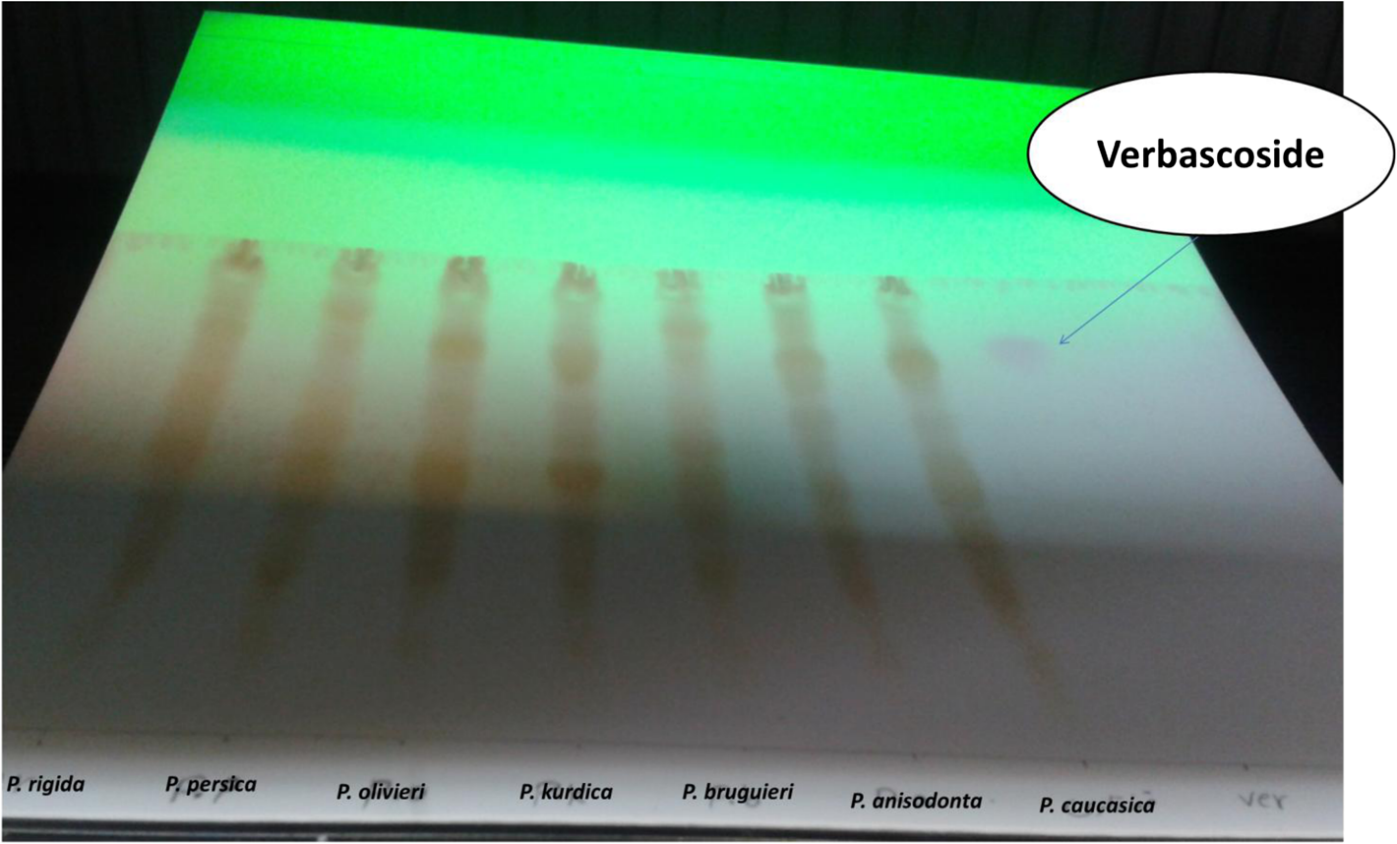
Verbascoside and the relative spots in the plant samples on a TLC plate under UV chamber (254 nm) in the mobile phase, ethylacetate: water: formic acid (10:3:2).

The specificity of the method was ascertained by comparing the Rf values and the spectrum of verbascoside standard with the spectrum obtained from a sample of the extract, at three different positions on the bands, i.e. peak start (S), peak apex (M), and peak end (E) [19]. The R*f* value for verbascoside and the relative spots in the plant samples was equal to 0.65 in the mobile phase as ethylacetate: water: formic acid, (10:3:2) (Figure 3).

## Results and discussion

**Figure 4:**
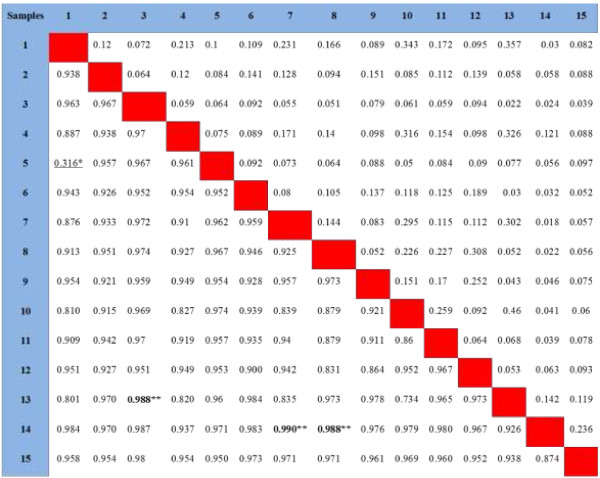
**Simple matching coefficient (S**_**sm**_**, above the diagonal) and genetic distances (d, below the diagonal) between pairs of *Phlomis* samples resulted from RAPD.** (1) *P*. olivieri (Kordestan), (2) *P*. persica (Kordestan), (3) *P*. rigida (kordestan), (4) *P*. kurdica (Kordestan), (5) *P*. persica (dehgolan), (6) *P*. bruguieri (Kordestan), (7) *P*. anisodonta (Kordestan), (8) *P*. caucasica (Ahar), (9) *P*. olivieri (Shabestar), (10) *P*. olivieri (Azerbayjan), (11) *P*. olivieri (Tabriz), (12) *P*. anisodonta (Mazandaran), (13) *P*. persica (Khorasan), (14) *P*. olivieri (Mazandaran), (15) *P*. anisodonta (Pole zangole); * The underlined number shows the closest genetic distance; ** The bold numbers exhibit the farthest genetic distances.

RAPD markers have been widely used in the analysis of genetic relationships and genetic diversity in a number of plant taxa because of its simplicity, speed and relatively low cost compared to other DNA-based markers. Pair-wise comparison of all RAPD profiles revealed a similarity matrix. Simple matching coefficients (Ssm) and genetic distances (d), derived from RAPD banding patterns, are shown in Figure 4. The range of genetic distances between different species of *phlomis* from Iran was calculated between 316-988.Actually, the most far away genetic distance (d = 0.990) has been observed between *P. bruguieri* (Kordestan) and *P. olivieri* from Mazandaran followed by the distance between *P. anisodonta* (Kordestan) and *P. persica* from Khorasan (d = 0.988), as well as *P. persica* (Kordestan) and *P. anisodonta* (Mazandaran) (d = 0.988), while the closest distance (d = 316) has been observed for *P. persica* from Dehgolan and *P. olivieri* from Kordestan. The farthest and closest distances are indicated in the Figure 4 by bold and underlined numbers, respectively. The genetic distance between the two samples, *P. olivieri* (Azerbayejan) and *P. persica* (Khorasan), was observed to be short (0.622) and their RAPD banding patterns were quite similar to each other; also there is a close relationship between these two samples of *Phlomis* with *P. olivieri* from Shabestar.

As shown in the dendrogram, various species of *P. olivieri* display close relationships to *P. anisodonta* and *P. persica*. On the other hand, *P. anisodonta* species represents the closest relationship with *P. Caucasica* and *P. kurdica*. It is interesting to note that different samples of *P. olivieri,* gathered from different habitats, did not exhibit so much close relationship to each other. Figure 2 shows a photo sample from a gel electrophoresis of all DNA samples alongside a DNA size marker in the presence of primer Zo21. RAPD molecular markers exhibited significant differences between various samples of the same species growing in different areas.

The results of quantification of verbascoside in different *Phlomis* species by using TLC scanner revealed that the highest concentration of verbascoside was found in *P. anisodonta*, however, *P. bruguieri* and *P. olivieri* (from Mazandaran) were in the second and third places (Table 3). Interestingly, the lowest concentration of verbascoside was detected in *P. olivieri* (from Azerbayjan), exhibiting the effect of various growing areas and conditions on the measured levels of this compound. Although the concentration of verbascoside in these plants may be affected by the growing areas and conditions, there are a good agreement between genetic relations and verbascoside levels. For instance, the concentration of verbascoside in three samples of *P.olivieri* (Azerbayjan, Shabestar, Tabriz) is significantly different (Table 3), alongside the far distances of these samples from the same species (Figure 1). Another example is almost equal level of verbascoside in *P. caucasica* and *P. olivieri* (Azerbayjan) (4.1 and 3.9 mg/mL, respectively) that support the close relationship between these two species (d = 0.879), in compared to those of Mazandaran and Kurdistan or Shabestar (Figure 4).

**Table 3:** **The concentration of verbascoside determined by using TLC-scanner in different species of *Phlomis***

**Plant Samples/or Standards**	**Calculated Area**	**Concentration (mg/mL) Mean ± SD**
***P. olivieri *** **(Azerbayjan)**	257559.8	3.9 ± 0.2
***P. olivieri *** **(Tabriz)**	461645.8	8.6 ± 0.2
***P. olivieri *** **(Mazandaran)**	479058.9	9.1 ± 0.4
***P. bruguieri***	506145.9	9.6 ± 0.5
***P. kurdica***	393148.1	7.0 ± 0.1
***P. rigida***	515808.8	9.8 ± 0.1
***P. anisodonta***	578081.9	11.3 ± 0.2
***P. persica***	463498.0	8.6 ± 0.4
***P. caucasica***	264396.1	4.1 ± 0.2
**Verbascoside (1.2 mg/mL)**	614373.5	12.0 ± 0.6
**Verbascoside (0.9 mg/mL)**	462891.9	9.0 ± 0.3
**Verbascoside (0.6 mg/mL)**	363006.8	6.0 ± 0.4
**Verbascoside (0.3 mg/mL)**	212619.3	3.0 ± 0.2

Verbascoside was previously isolated and identified from various species of *Phlomis*, of which *P. sieheana*, *P. samia*, *P. monocephala* and *P. carica* are recently reported from Turkey [20,21]. Furthermore, verbascoside is a phenylpropanoid glycoside well-known for its antioxidant, anti-inflammatory and photoprotective activity, and recently applied in dermocosmetic preparations. Moreover, verbascoside is used in formulation of suppositories for probable applications in treatment of inflammation in the intestinal mucosa [10,22]. A recent report reveals that verbascoside possesses stronger affinity for negatively charged membranes composed of phosphatidylglycerol than for phosphatidylcholine membranes. However, this compound can promot phase separation of lipid domains in phosphatidylcholine membranes and formed a stable lipid complex [23]. Regarding the importance of verbascoside as an active ingredient in *Phlomis* species, quantification of this compound is a successful method for standardization of the *Phlomis* extracts. Actually, the quantitative determination of phenylethanoid glycosides in methanolic extracts of five species of the genus *Phlomis* has been already investigated by using HPLC method combined with photodiode-array detection and electrospray/MS analysis. In that study, forsythoside B, verbascoside, samioside, alyssonoside, isoverbascoside, leucosceptosides A and B and martynoside were employed for detection. Although the results of that report is not comparable with the present study due to using different method of quantification as well as different species Phlomis, the investigators demonstrated that the content of phenylethanoid glycosides contributes to the chemotaxonomy of this genus [24]. Moreover, phenylethanoid glycosides like verbascoside, forsythoside B, and leucosceptoside A have been reported from *P. longifolia*[25]. However, acteoside and forsythoside B were also isolated from *P. tuberosa*[26].

Actually, plant secondary metabolite pathways have extensively been studied at the level of intermediates and enzymes that mainly lead to pharmaceutically important products. However, only a relatively small number of genes have been identified so far [27]. In fact, the idea about the probable link between plant genotype and its phytochemistry has been demonstrated by different studies so far. For instance, when genetic distance between genotypes of cottonwoods increased, the phytochemistry and arthropod community composition changed accordingly [28]. RAPD method, which can be used as one of the molecular biological techniques to determine genetic distances, has many advantages as follows: There is no need to knowledge of the DNA sequence for the targeted gene; It can be used for most genetic marker applications; The procedure is streamlined compared to other molecular analysis; It needs only thermocycler and agarose gel so is cost effective with less labor [29].

The present study demonstrates that RAPD analysis can be used beside phytochemical analysis of secondary metabolites in plants, due to the relationship between phytochemistry and genetic distances, and may create the useful fingerprints and molecular profiles to support phytochemical diversity data from plants.

## Conclusions

In conclusion, the present study reveals that verbascoside can be found in various species of Iranian *Phlomis*, of which *P. anisodonta*, *P. bruguieri* and *P. olivieri* might be the best choices. In addition, although the concentration of verbascoside in these plants may be affected by the growing areas and conditions, there are a good agreement between genetic relations and verbascoside levels.

## Competing interests

The authors declared that there is no conflict of interests.

## Authors’ contributions

PP: Interpreting of the HPTLC data; MN: DNA extraction and analysis; PP: Participating in TLC scanner analysis; ARG: Plant gathering and HPTLC advising; YA: Plant identification; SRH: Standardization of the HPTLC method; AH: Advising the plant extraction and verbascoside analysis; SS: Participating in manuscript drafting and interpreting of RAPD data. All authors read and approved the final manuscript.

## Supplementary Material

Additional file 1 **The number of unique bands in different samples of *Phlomis *** **produced by each primer.** Click here for file
