# Supplementary material for: Quantification of verbascoside in medicinal species of Phlomis and their genetic relationships
Source: Daru. 2014 Mar 20;22(1):32. doi: 10.1186/2008-2231-22-32 (PMC3998186; doi:10.1186/2008-2231-22-32)
Supplement: Additional file 1 — The number of unique bands in different samples of Phlomis produced by each primer. [file 2008-2231-22-32-S1.docx]

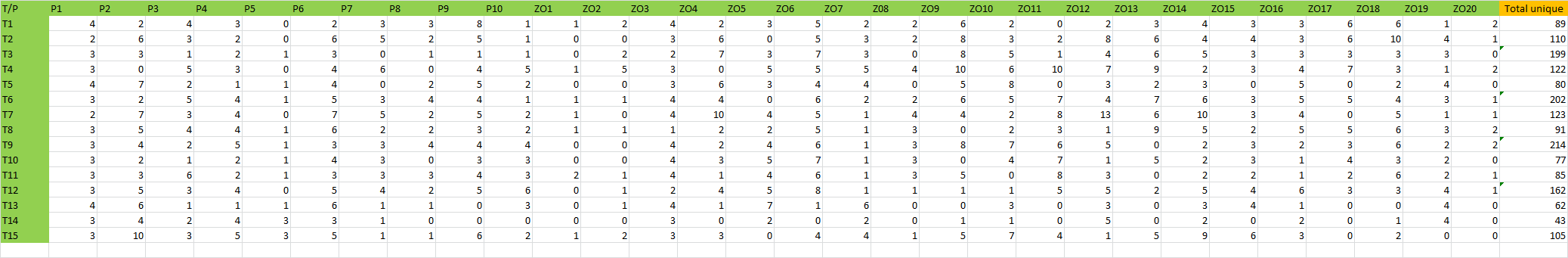


**Additional file 1:** The number of unique bands in different samples of *Phlomis* produced by each primer.

(1) P. olivieri-kor (Kordestan), (2) P. persica-kor (Kordestan), (3) P. rigida-kor (kordestan), (4) P. kurdica-kor (Kordestan) , (5) P. persica-dehgolan (kordestan) (6) P. bruguieri-kor (Kordestan), (7) P. anisodonta-kor (Kordestan), (8) P. caucasica (Ahar), (9) P. olivieri-shabestar (Shabestar), (10) P. olivieri-azer (Azerbayjan), (11) P. olivieri-tabriz (Tabriz), (12) P. anisodonta-maz (Mazandaran), (13) P. persica-khor (Khorasan), (14) P. olivieri-maz (Mazandaran), (15) P. anisodonta-siahbishe (Pole zangole).
